# Empty sella in somatotropic pituitary adenomas; a series of 23 cases

**DOI:** 10.3389/fsurg.2024.1350032

**Published:** 2024-03-04

**Authors:** Guive Sharifi, Esmaeil Mohammadi, Elham Paraandavaji, Seyed Mohammad Tavangar, Mohammad Amin Dabbagh Ohadi, Ali Jafari, Amin Jahanbakhshi, Nader Akbari Dilmaghani, Zahra Davoudi, Timothy R. Smith, Gelareh Banihashemi, Masoumeh Azadi, Neda Hatami, Georgios A. Zenonos, Mohammadreza Mohajeri Tehrani

**Affiliations:** ^1^ Department of Neurosurgery, Loghman Hospital, Shahid Beheshti University of Medical Science, Tehran, Iran; ^2^ Skull Base Research Center, Loghman Hospital, Shahid Beheshti University of Medical Sciences, Tehran, Iran; ^3^ Department of Neurosurgery, Tehran University of Medical Sciences, Tehran, Iran; ^4^ Department of Pathology, Shariati Hospital, Tehran University of Medical Sciences, Tehran, Iran; ^5^ Chronic Diseases Research Center, Endocrinology and Metabolism Population Sciences Institute, Tehran University of Medical Sciences, Tehran, Iran; ^6^ Department of Neurosurgery, Brigham and Women's Hospital, Harvard Medical School, Boston, MA, United States; ^7^ Department of Neurology, Sina Hospital, Tehran University of Medical Sciences, Tehran, Iran; ^8^ Endocrine Research Center, Institute of Endocrinology and Metabolism, Iran University of Medical Sciences, Tehran, Iran; ^9^ Department of Neurosurgery, University of Pittsburgh Medical Center, Pittsburgh, PA, United States; ^10^ Endocrinology and Metabolism Research Center, Endocrinology and Metabolism Clinical Sciences Institute, Tehran University of Medical Sciences, Tehran, Iran

**Keywords:** empty sella, acromegaly, adenoma, surgery, endoscopic surgery

## Abstract

**Purpose:**

We aimed to investigate empty sella syndrome in somatotrophic pituitary adenoma for possible etiology, complications, and treatment options.

**Method:**

Among over 2,000 skull base masses that have been managed in our center since 2013, we searched for growth hormone-producing adenomas. Clinical, surgical, and imaging data were retrospectively collected from hospital records to check for sella that lacked pituitary tissue on routine imaging.

**Result:**

In 220 somatotrophic adenomas, 23 patients had an empty sella with surgical and follow-up data. The mean age of the sample was 46 years with the same male-to-female ratio. Five cases had partial empty sella and the rest were complete empty sellas. The most common simultaneous hormonal disturbance was high prolactin levels. Six had adenoma invasion into the clivus or sphenoid sinus and 10 had cavernous sinus intrusion. Peri-operative low-flow and high-flow cerebrospinal fluid (CSF) leaks were encountered in one and two patients, respectively, which were successfully sealed by abdominal fat. The majority of cases required growth hormone replacement therapy while it was controlled without any replacement therapy in nine patients. No pituitary hormonal disturbance occurred after transsphenoidal surgery except for hypothyroidism in one patient.

**Conclusion:**

An empty sella filled with fluid can be detected frequently in pituitary adenomas, especially in the setting of acromegaly. The pituitary gland may be pushed to the roof of the sella and might be visible as a narrow rim on imaging or may be detected in unusual places out of the sella. The pathophysiology behind such finding originates from soft and hard tissue changes and CSF pressure alternations during abundant growth hormone production.

## Introduction

**Figure 1:**
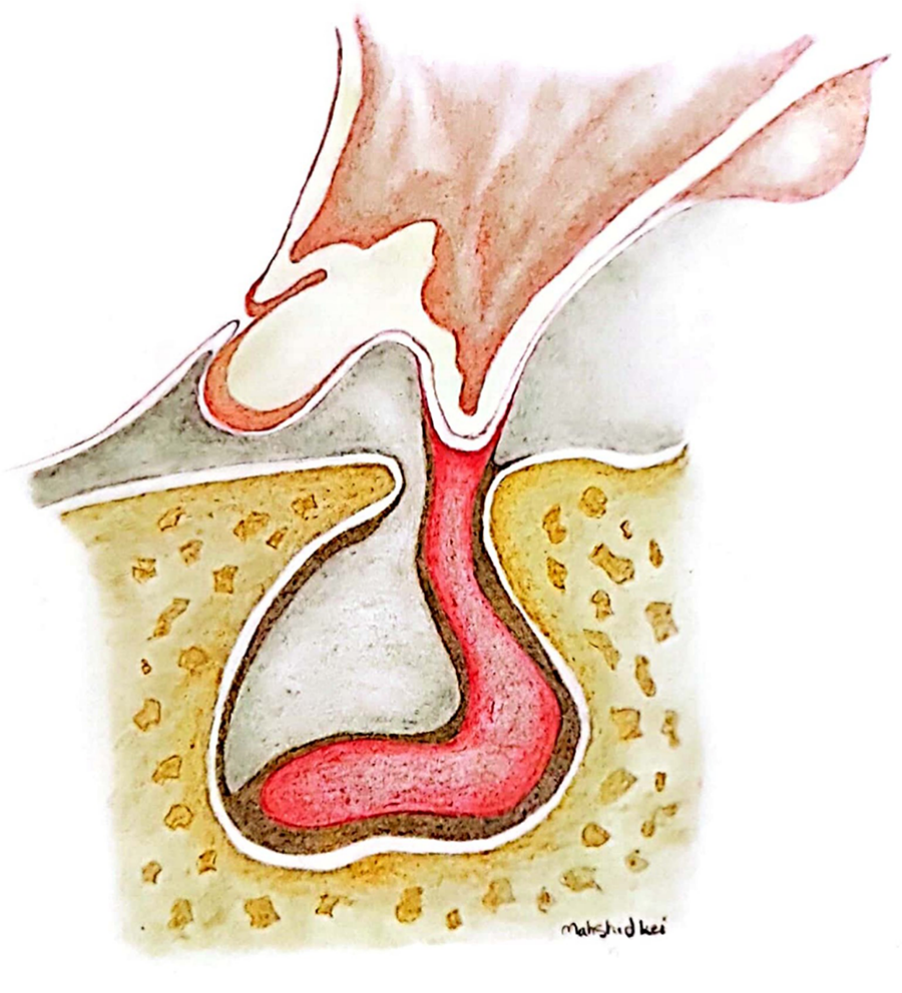
Anatomical illustration of the pushed-aside pituitary tissue by the arachnoid cyst in the context of empty sella.

Empty sella syndrome (ESS) is a neuro-radiological phenomenon defined by enlarged sella turcica filled with CSF and a flattened or pressed-aside pituitary (Figure 1) (1). Previous related radiotherapy, surgery, or trauma could lead to ESS with ascertained cause, better recognized as secondary empty sella. The primary or idiopathic form is rare and corroborated for those with no definitive reason (2, 3). An incompetent diaphragm, increased intracranial pressure, and pregnancy-associated changes in pituitary gland volume are among the proposed relatable factors.

Empty sella has been detected in ACTH and GH-secreting adenomas (2). Previous reports suggest higher rates of peri-operative cerebrospinal fluid (CSF) leakage and hormonal dysfunction in corticotropic adenomas and empty sella (4). Additionally, controversy exists around the role of transsphenoidal resection in acromegaly patients with coexisting empty sella (5).

Concomitance of empty sella and somatotrophic adenoma has been reported in prior small series and case reports while hormonal outcomes and long-term complications after surgery remain unclear. In this study, we report our experience with transsphenoidal surgery of GH-producing adenomas in a series of patients with coexisting empty sella and report procedural hardships and drawbacks.

## Method

### Patients

For this case series, we followed the PROCESS guideline (6) and retrospectively searched for empty sella cases in our database of more than 2,000 adenomas who underwent surgical resection between 2013 and 2020. All cases were operated on in a single tertiary academic center by the same surgery team. Prior to the surgery, patients were evaluated by an expert team including a neurologist who examined the patients, an endocrinologist who made the diagnosis of central acromegaly by established criteria (7), and a radiologist who evaluated the pituitary dynamic magnetic resonance imaging (MRI). Patients who had a previous history of interventional treatment (surgery or radiation therapy), known neurological disease, or GH/IGF1 (insulin-like growth factor) secreting ectopic tumors were excluded [using chest computed tomography (CT) scan]. We collected our data through hospital documents and follow-up of outpatient records. Further information was gathered via phone calls with the patients. All patients were followed up through routine clinic visits and perpetual imaging at the first month, sixth month, and then annual evaluations. Every enrolled participant was followed up for at least 3 years, post-operatively. Any record with a lack of surgical or medical data was excluded. No age or sex limit was placed in this work. The study design and conduct were in accordance with the Declaration of Helsinki (8) and our Institutional Review Board (IRB). The research was conducted using de-identified data following the acquisition of informed consent.

### Neuroimaging examination

Dynamic pituitary gland study MR images on a 1.5 T scanner with a standard head coil, along with axial and coronal non-contrast head CT scans were obtained preoperatively for all patients. Emptiness of sella was decided on midsagittal T1-weighted MR imaging (2). This was approved by an expert radiologist. Total empty sella was defined as a pituitary gland with less than 2.0-mm height or fluid filling of more than 50% of turcica's space, and partially empty sella was defined as a pituitary gland with a height greater than 2.1-mm or fluid filling between 50% and 33% of sellar space or its depth (9). The maximum tumor size (diameter) was measured on the coronal plane using a T1-weighted post-enhanced image. Tumors with a maximum diameter greater than 10 mm were categorized as macroadenomas (maximize area >100 mm^2^). Suprasellar extension of tumors was defined as growth toward the diaphragm sella or above the inferior optic chiasm plane. Extension to the left or right from the midline was also examined as a lateral extension of the tumoral tissue. Bony invasion was defined as a tumor growth in the sphenoid sinus documented with MRI or during the surgery.

### Operation

All patients underwent endonasal transsphenoidal surgery (TSS) for adenoma resection. All operations were performed by the lead surgeon (G.Sh) and the same team. Every specimen was sent to a pathologist for confirmation of GH-producing adenoma using immunohistochemistry. After tumor removal, due to the tendency of such adenomas to grow in extra-sellar locations, we thoroughly investigated different aspects and angles of sella to make sure no remnant was left. CSF leakage was classified as no observable leak, little amount of CSF leaks as a result of transient increase in intracranial pressure which is termed as a “low-flow” CSF leak, and “high-flow” continuous CSF leak observed intraoperatively in the setting of openings in the ventricular system or connections with cisterns (10, 11). In case of CSF leakage, abdominal fat and/or fascia were used as packing material for reconstruction.

### Endocrinology evaluation

Basal fasting GH, IGF1, and 75-gram oral glucose tolerance were assessed for patients before and after surgery. Other hormonal conditions were also recorded before surgery and followed up accordingly. Unfortunately, as the 75-gram oral glucose tolerance testing was not recorded for every patient, we excluded it from this report. We also reported coexisting hormonal problems.

## Results

### Case series

We found 31 somatotrophic adenomas with empty sella among 220 acromegaly patients who underwent transsphenoidal endoscopic surgery in our center. Eight records were excluded because of missing surgical characteristics. As a result, 23 patients were included. The mean age of the sample was 46 years and women accounted for 11/23 of the sample. The most prominent manifestation was an increase in the size of the hands, feet, and face in 19/23 patients. Other complaints were an increase in weight (more than 10 kg) in seven, headaches in six, and visual problems in five patients. One of the patients used octreotide and cabergoline, one used only octreotide, and another patient used levothyroxine before the surgery for less than 3 months.

### Surgical and anatomical result

**Figure 2:**
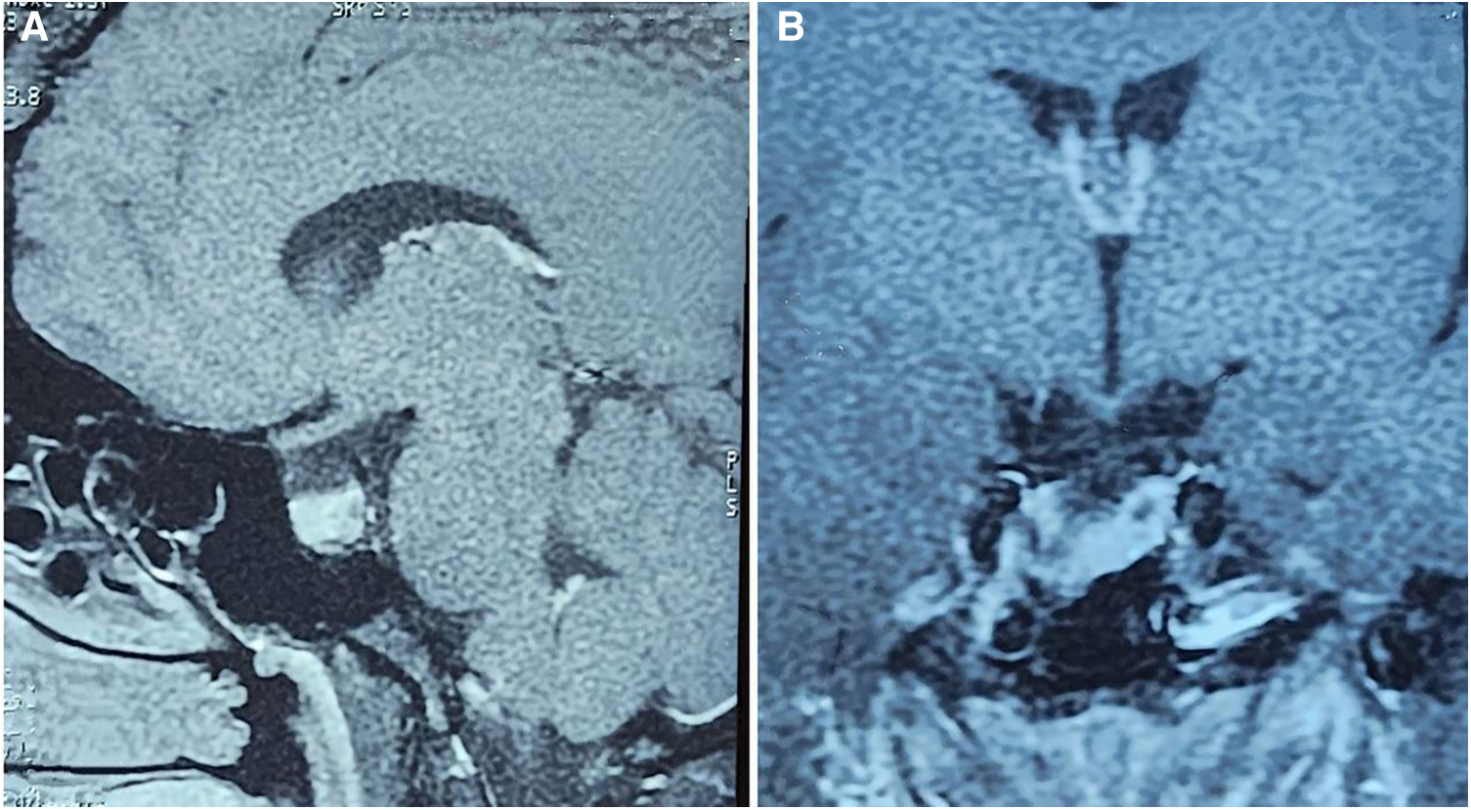
Sagittal (**A**) and coronal (**B**) views of dynamic MRI of the sellar region in patients with empty sella.

**Figure 3:**
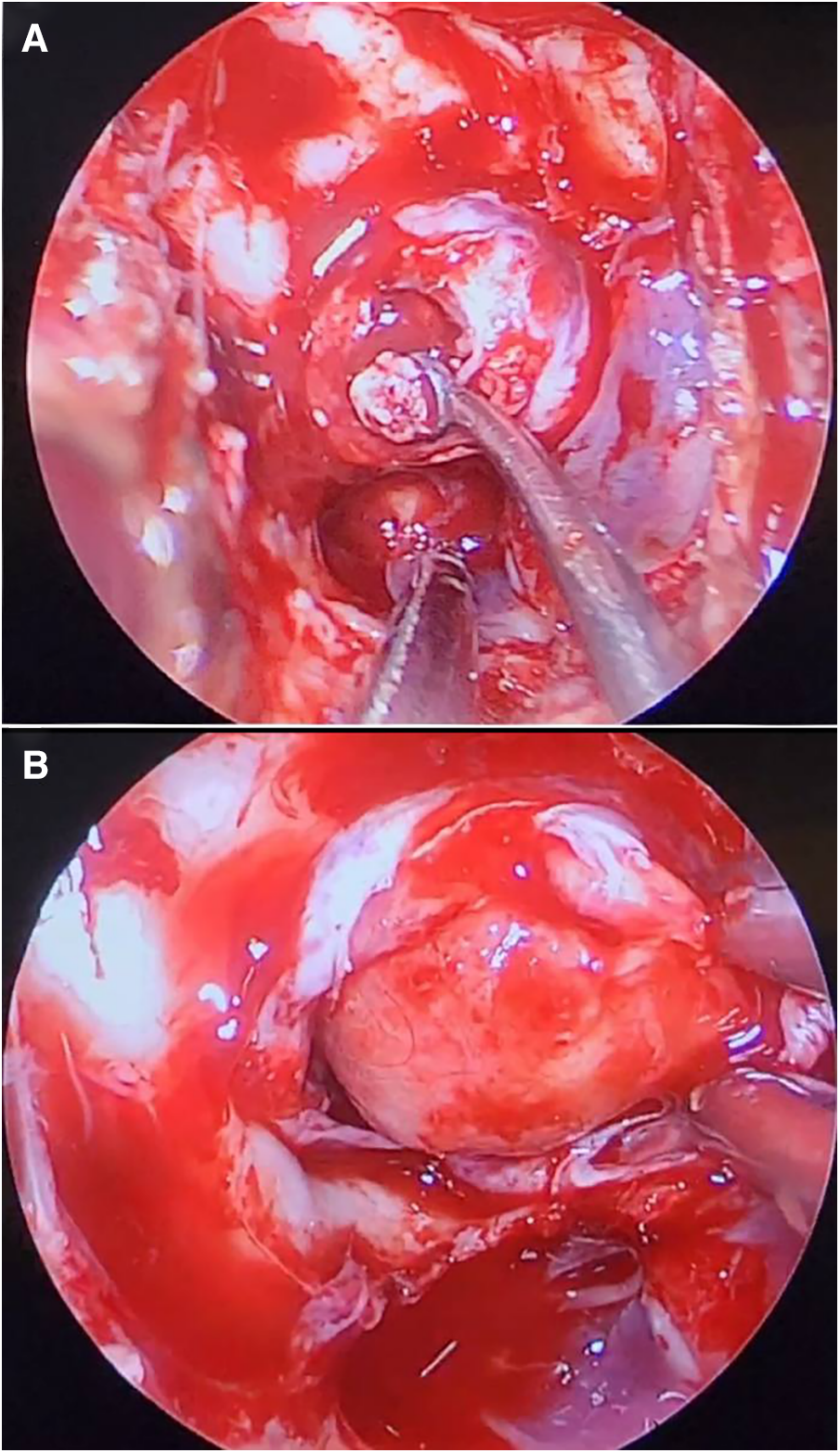
Endoscopic transnasal transsphenoidal surgery removal of a growth hormone-producing adenoma (**A**). Arachnoid extrusion is visible upon removal of sphenoid bone (**B**).

Preoperative MRI revealed 5/23 patients with partial empty sella and the rest with total empty sella (Figures 2, 3). Macroadenoma was discovered in 12 patients. The mean adenoma area was measured at 1.6 cm^2^. The pituitary gland was superiorly dislocated and visible as a rim of soft tissue in patients with total empty sella. The tumor was found to have an extension to suprasellar and parasellar regions in two and eight patients, respectively. Tumor invasion to the sphenoid sinus or clivus occurred in six patients. There was no evidence of apoplexy in any of the patients. During the surgery, low-flow and high-flow leaks were encountered in one and two patients, respectively, which were uneventfully sealed by abdominal fat. None of the patients reported postsurgical rhinorrhea or other signs of CSF leak during the follow-up. The CSF leakage rate was twice as high in this sample compared to the total case mix (4.7%). Further details are provided in Table 1.

**Table 1:** Surgical and anatomic findings.

Patients number	Age	Sex	ES type	Size of the adenoma	Cavernous (right, left, suprasellar extension)	Op CSF leak (none, low flow, high flow)	Invasion
#1	33	Male	Total	Micro	No	No	Yes
#2	57	Female	Total	Micro	No	No	Yes
#3	46	Female	Total	Macro	No	No	Yes
#4	66	Female	Partial	Macro	No	High flow	No
#5	46	Male	Partial	Macro	Right	High flow	No
#6	45	Male	Total	Micro	Left	No	No
#7	53	Female	Total	Micro	Left	No	No
#8	60	Female	Total	Micro	No	No	Yes
#9	61	Female	Total	Micro	Right	No	No
#10	36	Male	Total	Macro	Right	No	No
#11	34	Male	Total	Macro	Left	Low flow	No
#12	61	Male	Total	Macro	No	No	No
#13	50	Male	Total	Macro	Left	No	No
#14	31	Male	Partial	Micro	No	No	No
#15	32	Female	Total	Micro	No	No	No
#16	37	Female	Total	Micro	No	No	No
#17	55	Male	Total	Micro	Left	No	Yes
#18	37	Female	Partial	Macro	No	No	No
#19	45	Female	Total	Macro	Suprasellar	No	No
#20	54	Female	Total	Macro	No	No	No
#21	34	Male	Total	Macro	Suprasellar	No	Yes
#22	38	Male	Partial	Micro	No	No	No
#23	44	Male	Total	Macro	No	No	No

ES, empty sella; Op, operational.

### Endocrinology results

Pre-operative high prolactin levels were found in 4/23 patients as the most common simultaneous hormonal imbalance. Post-operative transient diabetes mellitus was found only in 1/23 patients which resolved over the follow-up period. Endocrinology results were available for 17 cases. At the last follow-up, normal GH and IGF1 were recorded in 9/17 patients without any medication. In addition, hormonal levels were controlled in 5/17 patients with medication after surgery, and 3/17 patients still had high levels of GH/IGF1. None of the patients reported any continuing or emergent symptoms after surgery except patient #9 who became hypothyroid. More details are provided in Table 2.

**Table 2:** Endocrinology findings pre-surgery and on follow-up.

Patients number	Pre OP GH (ng/ml)	Pre OP IGF (ng/ml)	Post OP GH (ng/ml)	Post OP IGF (ng/ml)	Other hormonal abnormality	Medication	Signs and symptoms before surgery
#1	3.6 (0.03–2.4)	602 (72–421)	0.45 (0–3)	227 (63–323)	High prolactin: reversed	Cabergoline levothyroxine	Increased size lumbosacral pain
#2	20.9	925 (204)	2.04 (0.07–8.3)	241 (54–240)	High TSH: reversed	–	Increased size
#3	33.13 (up to 12.4)	482 (60–271)	1.8 (0.32–5.06)	243 (35–245)	–	–	Increased size
#4	2.25 (0.1–10)	460 (69–200)	0.51 (0.06–6.88)	231.8 (54–204)	–	Levothyroxine	Increased size headache vision loss
#5	9.81 (0–4)	797 (95–165)	NL	NL	–	Levothyroxine	Increased size right hemiparesis
#6	8 (0–4)	483 (271–550)	NL	173 (86–196)	–	–	Increased size headache
#7	9 (0–18)	746 (0–160)	0.52 (0–18)	181.6 (54–204)	–	–	Increased size headache sweating hyperglycemia increased weight
#8	1.2 (0–8)	323 (43–241)	NL	NL	Transient diabetes insipidus	Cabergoline	Increased weight blurry vision
#9	11 (0.12–10)	425.5 (29–204)	4.3 (0–1.23)	120 (0–244)	High Prolactin: Reversed High FSH: Reversed low TSH after surgery	Sandostatin LAR levothyroxine	Increased size
#10	3.1 (0.02–1.23)	1,032 (82–242)	0.31 (0–2.47)	230 (83–232)	–	Cabergoline	Increased weight increased size
#11	21 (0.2–4.7)	1,321 (115–370)	0.51 (0.02–4.77)	642 (96–227)	High Prolactin: Reversed	Sandostatin LAR cabergoline DDAVP nasal spray	Headache increased weight increased size blurry vision
#12	High	569 (106–546)	0.88 (<9)	128 (<216)	Low TSH before and after Surgery	Levothyroxine	Increased size increased weight blurry vision
#13	7.7 (0–2.5)	398 (69–343)	0.1 (0–2.5)	185 (97–292)	–	–	Increased size
#14	8.92 (0–2)	501.6 (87–415)	0.31 (0–66)	280.9 (115–230)	–	–	Headache increased weight increased Size
#15	10.73	299	0.48 (0.1–10)	195 (73–243)	High prolactin: reversed	–	Increased size reduced hearing blurry vision
#16	6.9 (0.06–6.8)	370 (67–230)	1.46 (0.1–10)	222.9 (69–227)	–	Sandostatin LAR	Paresthesia Increased size
#17	1.95 (0–5)	168 (97–292)	0.03 (0–66)	177 (97–292)	–	–	Headache increased weight increased size

NL, normal.

## Discussion

Here we stated our experience of pituitary surgery of somatotrophic adenomas with concomitant empty sella syndrome. Approximately 14% of GH-producing adenomas were detected to present with a sella filled with fluid. This entity has been observed in a wide range from 15% to much higher rates, as Bjerr et al. (12) encountered empty sella in half of their acromegaly patients. Some believe empty sella is an incidental finding in acromegaly adenoma situation (5) while others justify this coexistence. Liu et al. proposed empty sella coexistence with acromegaly relatable to the paracrine effects of GH (2). In other words, due to the anabolic effects of GH on soft and hard tissues, it has been stipulated that the sellar anatomy of acromegaly cases might be larger. Similarly, it is shown that in such instances the diaphragm of sella is firmer and less compliant, making pituitary tissue and adenomas more susceptible to growing to the extra-sellar areas. Empty sella is a known sequel to idiopathic intracranial hypertension (IIH), which is more prevalent in women (7). On the other hand, GH is a predisposing element for IIH likely due to association with higher levels of α-Klotho, an important protein in CSF homeostasis (2, 13). However, mechanistic studies are required to evaluate this association. Adenoma infraction and hemorrhage are among other implausible reasons for ESS, but they were not observed in any of the enrolled participants.

High levels of prolactin were the most common coexisting hormonal problem followed by abnormal levels of TSH. Additionally, for ESS differential diagnosis, ectopic GH/IGF1 secreting adenoma in the bronchus and sphenoid sinus should be excluded (14, 15).

Despite inconsistencies in surgical outcomes, TSS remains the gold standard treatment for adenomas with coexisting empty sella although a great deal of attention should be paid to explore sella's roof as well as higher extra-sellar regions (5). High levels of prolactin were the most common coexisting hormonal problem followed by abnormal levels of TSH. GH/IGF1 levels were controlled without any medications in 53% of the cases with other hormones adjusting with it. This was similar to previous studies, interpretable as half of the cases will be curable with no need for hormonal regimens (2, 5). However, cases with empty sella may require hormonal therapies to a higher extent than regular adenomas that could maintain the hormonal needs with no further assistance (50%–70%) (16, 17). Anterior pituitary malfunction and diabetes insipidus after surgery were seen in a previous study by Wang et al. in 18% and 37% of cases, respectively. Nevertheless, this complication occurred only in one of our patients. Few studies have mentioned risk factors for poor surgical outcomes (5, 18). Complete emptiness of sella has been referred to as a major risk factor for a poorer cure rate (5, 18). Higher GH levels and larger adenomas are other suggested risk factors for poor outcomes (18, 19). CSF leakage was the most frequent complication of surgery in our sample. CSF leakage is a common complication after TSS which could potentially lead to infection and neurologic complications (20, 21). CSF leakage in empty sella entity was twice higher compared to our total case mix, possibly due to higher intracranial pressure. Similar findings in acromegaly patients with empty sella have been found in prior works (5). Wang et al. showed this complication to be related to larger tumors accompanying empty sella (18). However, in certain cases with high suspicion of tumor remnant, it might be decided to open the occupying arachnoid cyst in order to ease access to different aspects of the sellar region. However, the use of fat tissue extracted from the abdomen for the reconstruction of defects and leaks in our sample has been found to be effective and is the readily available method (22).

Despite the efforts to collect comprehensive information, our study was limited by missing data. Mechanistic investigations are needed to clarify the exact pathophysiology behind the tendency of arachnoid cysts and empty sella with various adenoma subtypes.

In conclusion, sella occupied with arachnoid is a coexisting feature of a considerable number of somatotropic pituitary adenomas, a higher rate than what is previously stipulated, which could push aside the pituitary tissue to the margins of sella or upper locations. Although the exact etiology of this entity remains unclear, coexisting idiopathic intracranial hypertension as well as anatomical changes due to growth hormone overexpression and diaphragm incompliance could lead to displaced adenoma. Appropriate preoperative imaging and thorough evaluation of the sella region are advised in suspected cases. Transsphenoidal surgery provides satisfactory results and infrequent complications with a comparable number of regular adenomas without empty sella. The most frequently encountered complicating event is cerebrospinal fluid leakage which could be managed at the same success rate as other skull-based surgeries.

## Data Availability

The deidentified data supporting the conclusions of this article will be made available by the authors, without undue reservation.
